# Supplementary material for: Spatial distribution and determinants of abortion among reproductive age women in Ethiopia, evidence from Ethiopian Demographic and Health Survey 2016 data: Spatial and mixed-effect analysis
Source: PLoS One. 2020 Jun 29;15(6):e0235382. doi: 10.1371/journal.pone.0235382 (PMC7323954; doi:10.1371/journal.pone.0235382)

PLOS ONE

Spatial distribution and determinants of abortion among reproductive-age women in Ethiopia, Evidence from Ethiopian Demographic and Health Survey (EDHS) 2016 data: Spatial and Mixed-effect analysis

--Manuscript Draft--

**Manuscript Number:**	PONE-D-19-30532
**Article Type:**	Research Article
**Full Title:**	Spatial distribution and determinants of abortion among reproductive-age women in Ethiopia, Evidence from Ethiopian Demographic and Health Survey (EDHS) 2016 data: Spatial and Mixed-effect analysis
**Short Title:**	Spatial distribution and determinants of abortion among reproductive-age women in Ethiopia
**Corresponding Author:**	Getayeneh Antehunegn Tesema, Masters degree<br>University of Gondar College of Medicine and Health Sciences<br>Gondar, Ethiopia ETHIOPIA
**Keywords:**	Abortion; mixed-effect logistic regression; spatial analysis; Ethiopia
**Abstract:**	Background<br><br>Abortion is the major cause of maternal mortality in developing countries. Despite the efforts made to reduce maternal mortality in the last decades, abortion ~~yet constitutes~~ the highest maternal mortality in Ethiopia. So far, data on spatial distribution and factors determining abortion in the country is limited. This study aimed to explore the spatial pattern and determinants of abortion among women aged 15-49 years in Ethiopia, 2016.Methods<br><br>Secondary data analysis was conducted based on the 2016 Ethiopian Demographic and Health Survey (EDHS) data. Weighted samples of 12,378 women were included in the study. ~~The~~ Bernoulli model has been fitted using spatial scan statistics version 9.6 to identify hotspot areas and ArcGIS version 10.6 to explore the spatial distribution of abortion. For determinants, a mixed effect logistic regression model that accounts for the hierarchical structure of the data was fitted using STATA version 14.Result<br><br>The study showed that the prevalence of abortion in Ethiopia was 8.9% [95% CI: 8.4%, 9.5%], which was widely varied across regions ranging from 4.5% in Benishangul to 11.3% in Tigray regions. The spatial distribution of abortion was non-random in Ethiopia. The spatial scan statistics identified a total of 60 significant clusters within 4 spatial windows; of which 19 clusters were primary clusters (most likely clusters). The primary clusters were located in the northern part of the Tigray region (LLR=26.6, p<0.01; RR=2.63). In the final model of multivariable mixed-effect logistic regression analysis, primary education [AOR=1.36; 95% CI(1.13, 1.64)], rural residence [AOR=4.96; 95%CI(3.42, 7.18)], protestant religion [AOR=0.56; 95%CI: 0.42, 0.75)], richest wealth status [AOR=1.72; 95% CI(1.24, 2.40)], maternal age 24-19 [AOR=2.20; 95%CI(1.27, 3.80)], 30-34 [AOR=3.2; 95%CI(1.82, 5.71)], 35-39 [AOR=3.01; 95%CI(1.67, 5.42)], 40-44 [AOR=4.57; 95%CI(2.47, 8.46)] and 45-49 years (AOR=3.12; 95%CI(1.52, 6.44)], listening radio [AOR=1.27; 95%CI(1.01, 1.60)] and watching TV [AOR=1.45; 95%CI: 1.04, 2.01)] were significantly associated with abortion.Conclusions<br><br>Abortion spatial distribution among reproductive-age women in Ethiopia was not random, with significant hotspots of higher prevalence of abortion in the northern parts of Tigray, border areas between Oromia and Amhara regional states, border areas between Oromia, and SNNP regions. Maternal education, residence, media exposure, wealth status, and religion were significant predictors. The regions with higher hotspot areas of abortion would better target socioeconomic factors including maternal education, residence, media exposure, wealth status, and religion to curtail the prevalence of abortion.
**Order of Authors:**	Getayeneh Antehunegn Tesema, Masters degree

Powered by Editorial Manager® and ProduXion Manager® from Aries Systems Corporation

	Tesfaye Hambisa Mekonnen
	Achamyeleh Birhanu Teshale
**Opposed Reviewers:**	
**Additional Information:**	
**Question**	**Response**
**Financial Disclosure**<br><br>Enter a financial disclosure statement that describes the sources of funding for the work included in this submission. Review the submission guidelines for detailed requirements. View published research articles from *PLOS ONE* for specific examples.<br><br>This statement is required for submission and **will appear in the published article** if the submission is accepted. Please make sure it is accurate.<br><br>**Unfunded studies**<br>Enter: *The author(s) received no specific funding for this work.*<br><br>**Funded studies**<br>Enter a statement with the following details:<br>• Initials of the authors who received each award<br>• Grant numbers awarded to each author<br>• The full name of each funder<br>• URL of each funder website<br>• Did the sponsors or funders play any role in the study design, data collection and analysis, decision to publish, or preparation of the manuscript?<br>• **NO** - Include this sentence at the end of your statement: *The funders had no role in study design, data collection and analysis, decision to publish, or preparation of the manuscript.*<br>• **YES** - Specify the role(s) played.<br><br>* typeset	N/A
**Competing Interests**<br><br>Use the instructions below to enter a competing interest statement for this submission. On behalf of all authors, disclose any competing interests that could be perceived to bias this	The authors have declared that no competing interests exist

work—acknowledging all financial support and any other relevant financial or non-financial competing interests.<br><br>This statement **will appear in the published article** if the submission is accepted. Please make sure it is accurate. View published research articles from *PLOS ONE* for specific examples.<br><br>**NO authors have competing interests**<br><br>Enter: *The authors have declared that no competing interests exist.*<br><br>**Authors with competing interests**<br><br>Enter competing interest details beginning with this statement:<br><br>*I have read the journal's policy and the authors of this manuscript have the following competing interests: [insert competing interests here]*<br><br>* typeset	
**Ethics Statement**<br><br>Enter an ethics statement for this submission. This statement is required if the study involved:<br><br>• Human participants<br>• Human specimens or tissue<br>• Vertebrate animals or cephalopods<br>• Vertebrate embryos or tissues<br>• Field research<br><br>Write "N/A" if the submission does not require an ethics statement.<br><br>General guidance is provided below. Consult the submission guidelines for detailed instructions. **Make sure that all information entered here is included in the Methods section of the manuscript.**	N/A

**Format for specific study types**<br><br>**Human Subject Research (involving human participants and/or tissue)**<br>• Give the name of the institutional review board or ethics committee that approved the study<br>• Include the approval number and/or a statement indicating approval of this research<br>• Indicate the form of consent obtained (written/oral) or the reason that consent was not obtained (e.g. the data were analyzed anonymously)<br><br>**Animal Research (involving vertebrate animals, embryos or tissues)**<br>• Provide the name of the Institutional Animal Care and Use Committee (IACUC) or other relevant ethics board that reviewed the study protocol, and indicate whether they approved this research or granted a formal waiver of ethical approval<br>• Include an approval number if one was obtained<br>• If the study involved *non-human primates*, add *additional details* about animal welfare and steps taken to ameliorate suffering<br>• If anesthesia, euthanasia, or any kind of animal sacrifice is part of the study, include briefly which substances and/or methods were applied<br><br>**Field Research**<br><br>Include the following details if this study involves the collection of plant, animal, or other materials from a natural setting:<br>• Field permit number<br>• Name of the institution or relevant body that granted permission	
**Data Availability**<br><br>Authors are required to make all data underlying the findings described fully available, without restriction, and from the time of publication. PLOS allows rare exceptions to address legal and ethical concerns. See the PLOS Data Policy and FAQ for detailed information.	Yes - all data are fully available without restriction

A Data Availability Statement describing where the data can be found is required at submission. Your answers to this question constitute the Data Availability Statement and **will be published in the article**, if accepted.<br><br>**Important:** Stating 'data available on request from the author' is not sufficient. If your data are only available upon request, select 'No' for the first question and explain your exceptional situation in the text box.<br><br>Do the authors confirm that all data underlying the findings described in their manuscript are fully available without restriction?	
**Describe where the data may be found in full sentences. If you are copying our sample text, replace any instances of XXX with the appropriate details.**<br><br>• If the data are **held or will be held in a public repository**, include URLs, accession numbers or DOIs. If this information will only be available after acceptance, indicate this by ticking the box below. For example: *All XXX files are available from the XXX database (accession number(s) XXX, XXX.).*<br>• If the data are all contained **within the manuscript and/or Supporting Information files**, enter the following: *All relevant data are within the manuscript and its Supporting Information files.*<br>• If neither of these applies but you are able to provide **details of access elsewhere**, with or without limitations, please do so. For example:<br><br>*Data cannot be shared publicly because of [XXX]. Data are available from the XXX Institutional Data Access / Ethics Committee (contact via XXX) for researchers who meet the criteria for access to confidential data.*<br><br>*The data underlying the results presented in the study are available from (include the name of the third party*	the data available from dhs data bases

and contact information or URL).<br>• This text is appropriate if the data are owned by a third party and authors do not have permission to share the data.<br><br>* typeset	
Additional data availability information:	

Date: 1 November 2019

To: PLOS ONE Journal editors

Subject: **Manuscript submission for peer review and publication**

Dear Editor-in-Chief,

Please find enclosed our manuscript," *Spatial distribution and determinants of abortion among reproductive-age women in Ethiopia, Evidence from Ethiopian Demographic and Health Survey (EDHS) 2016 data: Spatial and Mixed-effect analysis* "by Getayeneh Antehunegn Tesema, Tesfaye Hambisa Mekonnen, and Achamyeleh Birhanu Teshale, which we would like to submit for publication as an original research in PLOS ONE. To our knowledge, this is the first report showing *spatial distribution and determinants of abortion among reproductive-age women in Ethiopia, Evidence from Ethiopian Demographic and Health Survey (EDHS) 2016 data: Spatial and Mixed-effect analysis in Ethiopia*. We believe our findings would appeal to the readership of PLOS ONE. We confirm that this manuscript has not been published elsewhere and is not under consideration by another journal. All authors have approved the manuscript and agree with its submission to PLOS ONE.

We look forward to hearing from you at your earliest convenience.
With best regards,

Sincerely yours,

Getayeneh Antehunegn Tesema

Correspondence

Email address: getayenehantehunegn@gmail.com

Gondar, Ethiopia

Manuscript
Click here to download Manuscript Abortion FV.docx

Spatial distribution and determinants of abortion among reproductive-age women in Ethiopia, Evidence from Ethiopian Demographic and Health Survey (EDHS) 2016 data: Spatial and Mixed-effect analysis

Getayeneh Antehunegn Tesema[1*], Tesfaye Hambisa Mekonnen[2], Achamyeleh Birhanu Teshale[1]

Affiliations:

[1]Department of Epidemiology and Biostatistics, Institute of Public Health, College of Medicine and Health Sciences, University of Gondar, Gondar, Ethiopia

[2]Department of Environmental and Occupational Health and Safety, Institute of Public Health, College of Medicine and Health Sciences, University of Gondar, Gondar, Ethiopia

Email addresses:

Getayeneh Antehunegn Tesema: getayenehantehunegn@gmail.com

Tesfaye Hambisa Mekonnen: tajan2022@gmail.com

Achamyeleh Birhanu Teshale: achambir08@gmail.com

[*]Correspondence

ABSTRACT

Background: Abortion is the major cause of maternal mortality in developing countries. Despite the efforts made to reduce maternal mortality in the last decades, abortion yet constitutes the highest maternal mortality in Ethiopia. So far, data on spatial distribution and factors determining abortion in the country is limited. This study aimed to explore the spatial pattern and determinants of abortion among women aged 15-49 years in Ethiopia, 2016.

Methods: Secondary data analysis was conducted based on the 2016 Ethiopian Demographic and Health Survey (EDHS) data. Weighted samples of 12,378 women were included in the study. The Bernoulli model has been fitted using spatial scan statistics version 9.6 to identify hotspot areas and ArcGIS version 10.6 to explore the spatial distribution of abortion. For determinants, a mixed effect logistic regression model that accounts for the hierarchical structure of the data was fitted using STATA version 14.

Result: The study showed that the prevalence of abortion in Ethiopia was 8.9% [95% CI: 8.4%, 9.5%], which was widely varied across regions ranging from 4.5% in Benishangul to 11.3% in Tigray regions. The spatial distribution of abortion was non-random in Ethiopia. The spatial scan statistics identified a total of 60 significant clusters within 4 spatial windows; of which 19 clusters were primary clusters (most likely clusters). The primary clusters were located in the northern part of the Tigray region (LLR=26.6, p<0.01; RR=2.63). In the final model of multivariable mixed-effect logistic regression analysis, primary education [AOR=1.36; 95% CI(1.13, 1.64)], rural residence [AOR=4.96; 95%CI(3.42, 7.18)], protestant religion [AOR=0.56; 95%CI: 0.42, 0.75)], richest wealth status [AOR=1.72; 95% CI(1.24, 2.40)], maternal age 24-19 [AOR=2.20; 95%CI(1.27, 3.80)], 30-34 [AOR=3.2; 95%CI(1.82, 5.71)], 35-39 [AOR=3.01; 95%CI(1.67, 5.42)], 40-44 [AOR=4.57; 95%CI(2.47, 8.46)] and 45-49 years (AOR=3.12; 95%CI(1.52, 6.44)], listening radio [AOR=1.27; 95%CI(1.01, 1.60)] and watching TV [AOR=1.45; 95%CI: 1.04, 2.01)] were significantly associated with abortion.

Conclusions: Abortion spatial distribution among reproductive-age women in Ethiopia was not random, with significant hotspots of higher prevalence of abortion in the northern parts of Tigray, border areas between Oromia and Amhara regional states, border areas between Oromia,

and SNNP regions. Maternal education, residence, media exposure, wealth status, and religion were significant predictors. The regions with higher hotspot areas of abortion would better target socioeconomic factors including maternal education, residence, media exposure, wealth status, and religion to curtail the prevalence of abortion.

Keywords: Abortion, mixed-effect logistic regression, spatial analysis, Ethiopia

Introduction

Abortion is defined as the loss of product of conception before the fetus surviving out of uretro (before 28 completed weeks of gestation) whether induced or spontaneous with its outcome safe or unsafe (1, 2). It is the major cause of maternal mortality among reproductive-age women (3). Annually, over 55.9 million abortions occur worldwide with 49.3 million in developing and 6.6 million in developed regions (4). Globally, abortion accounts for 13% of all maternal mortality (5), representing 200,000 maternal deaths and 5 million disabilities annually (6, 7). The majority of abortion-related mortality and morbidity can be prevented through education on sexual behavior, family planning and provision of safe abortion (8).

Abortion remains a serious public health concern(4) mainly in developing countries where unintended pregnancies often result from ineffective use or nonuse of contraceptives (9). The rate of abortion varies across countries, ranging from 31 per 1,000 women of reproductive age in western Africa to 38 per 1,000 in northern Africa rates in Eastern, Middle, and Southern Africa is close to the regional average of 34 per 1,000 (10, 11).

The rate of abortion has ~~been~~ declined in developed nations where the liberalization of abortion law and safe abortion service is legally available (12, 13). The prevalence however remains high in developing countries particularly Sub-Saharan Africa (SSA) where abortion is legally highly restricted (14-16).

Prior studies have documented that abortion has been an important and ongoing health problem in Ethiopia. In 2008, an estimated 382,000 induced abortions were performed and 52,600 women were treated for the complication of unsafe abortion with the prevalence of abortion of 13% (6). Ethiopian Demographic and Health Survey (EDHS) 2016 report estimates that 412 women died of pregnancy-related causes for every 100,000 live births in the five years before the survey(17). A study conducted in Southern Ethiopia showed that the prevalence of abortion mainly related to unwanted pregnancies was 12.3% (18).

Different studies done on abortion showed that rural residence, parity, educational status, mode of delivery, antenatal care (ANC) utilization, and place of delivery, maternal nutritional status, and maternal obstetric factors were the significant predictors of abortion (19-21).

There is a wide gap in abortion not only among different countries but also within the country (22). A high rate of stillbirth has been reported among rural, poor and marginalized societies that are the least beneficial of maternal health promotion activities and services(23, 24). Thus, the identification of geographic areas with a high rate of abortion using Geographic Information System (GIS) and Spatial Scan statistical analysis (SaTScan) has become fundamental to guide targeted public health interventions (25).

Although abortion is an acute public health problem in SSA including Ethiopia (26), information is scant on the spatial distribution and its determinant factors and few available data is not nationally representative. Hence, this study explored the spatial pattern and determinants of abortion among reproductive-age women in Ethiopia using the 2016 EDHS data. As abortion and abortion-related mortality is availability and quality indicator of maternal health services (27), understanding the significant hotspot areas of abortion is essential to evaluate the quality of service and improve access to maternal health services that in turn reduces maternal mortality.

Method and materials

Study design, setting and period

A community-based cross-sectional study was conducted from January 18 to June 27, 2016. The study was conducted in Ethiopia, which is situated in the Horn of Africa. The country has 9 National Regional States including Afar, Amhara, Benishangul-Gumuz, Gambela, Harari, Oromia, Somali, Southern Nations, Nationalities, and People's (SNNP) Region and Tigray and two Administrative cities (Addis Ababa and Dire-Dawa) **(Figure 1)**. Ethiopia is an agrarian country and agriculture accounts for 43% of the gross domestic product (GDP) and 84% of the population lives in rural areas. More than 80% of the country's total population lives in the regional states of Amhara, Oromia, and SNNP (28).

In 2016, the total population was102 million of which 43.47% of the population ages less than 14 years with a birth rate of 36.5 births per 1000 populations and fertility rate of 4.46. Ethiopia is the13th in the world and 2nd in Africa most populous country Ethiopia has 3 tiers health systems, Primary health care unit (Primary hospital, health center, health post, primary clinic, and medium clinic); Secondary health care (General hospital, specialty clinics and specialty centers); and Tertiary health care (Specialized hospital). The number of hospitals varies from

region to region in response to differences in population size. The most populous region, Oromia has 30 hospitals. The other two predominant regions Amhara and SNNP have 19 and 20 respectively with Tigray in fourth place with 16 hospitals, Gambela has only one hospital and Benishangul-Gumuz two had two hospitals (29).

Sample and population

The source population was all reproductive-age women within five years before the survey in Ethiopia and all women from reproductive-age women in the selected enumeration areas within five years before the survey were the study population. The EDHS used a stratified two-stage cluster sampling technique selected in two stages using the 2007 Population and Housing Census as a sampling frame. Stratification was achieved by ~~separating~~ each region into urban and rural areas. In total, 21 sampling strata have been created because the Addis Ababa region is entirely urban. In the first stage, 645 enumeration areas (EAs) (202 in the urban area) were selected with probability proportional to the EA size and with independent selection in each sampling stratum. In the second stage, because the time has passed since the population and housing census, a complete household listing operation was carried out in all selected EAs before the start of fieldwork and on average 28 households were systematically selected. Of these, 18,008 households and 16,583 eligible women included. The detailed sampling procedure was presented in the full EDHS 2016 report (30).

Variables and data collection procedure

The dependent variables used for the study was "pregnancy termination" or "abortion" which was derived from the EDHS question "have you ever had a terminated pregnancy" and then we ~~extract~~ terminated pregnancy within 5 years preceding the survey and the outcome variable was coded as 0= "No", if women didn't have terminated pregnancy within the study period, and as 1= "yes" if a women had pregnancy termination (abortion). The independent factors were maternal age, residence, educational status, marital status, religion, and frequency of watching television, frequency of listening radio, wealth status, and birth history.

The study was conducted based on EDHS data by accessing from the DHS program official database www.measuredhs.com after permission was granted through an online request by

explaining the objective of the study. A structured and pre-tested questionnaire was used as a tool for data collection. The raw data was collected from all parts of the country on childbearing aged women. We used the EDHS 2016 birth data set and extracted the outcome and independent variables. Geographic coordinate data (longitude and latitude coordinates) was taken at the cluster level/ enumeration area level after we explain the purpose of conducting the spatial distribution of abortion.

Data management and analysis

Before the actual data collection, data collectors were trained and a pretest was conducted. Interviews were performed using three local languages (Amharic, Tigrigna and Afaan Oromo). The data were weighted using sampling weight, primary sampling unit, and strata before any statistical analysis to restore the representativeness of the survey and to ~~tell the STATA to~~ take ~~into account the~~ sampling design when calculating standard errors to get reliable statistical estimates. Cross tabulations and summary statistics were done using STATA version 14 software. In EDHS data, the data structure is hierarchical nature, women are nested within a cluster and we expect that women within the same cluster may be more similar to each other than women in the rest of the country. This violates the assumption of the traditional regression model which is the independence of observations and equal variance across clusters. It implies that the need to take into account the between cluster variability by using advanced model. Therefore, a mixed effect logistic regression model (both fixed and random effect) was fitted. Since the outcome variable was binary standard logistic regression and Generalized Linear Mixed Models (GLMM) were fitted. Model comparison was done based on Intra-Class Correlation Coefficient (ICC), Likelihood Ration (LR) test, Akakie Information Criteria (AIC), Deviance and Bayesian Information Criteria (BIC) values. The model with the lowest deviance was chosen since the model is a nested model. Variables with <0.2 p-values in the bi-variable analysis were considered in the multivariable mixed-effect logistic regression model. Adjusted Odds Ratio (AOR) with a 95% Confidence Interval (CI) and p-value <0.05 in the multivariable model were used to declare significant association with abortion. The goodness of fit was assessed using deviance, LR test, and ICC.

Spatial analysis

For the spatial analysis ArcGIS version 10.6 software and SaTScan version 9.6 software have been used.

Spatial autocorrelation analysis

The spatial autocorrelation (Global Moran's I) statistic measures whether abortion patterns were dispersed, clustered or randomly distributed in the study area (31). Moran's I is a spatial statistics used to measure spatial autocorrelation by taking the entire data set and produce a single output value which ranges from -1 to +1. Moran's I Values close to −1 indicate disease dispersed, whereas Moran's I close to +1 indicate disease clustered and disease distributed randomly if I value is zero. A statistically significant Moran's I ($p < 0.05$) leads to rejection of the null hypothesis (abortion is randomly distributed) and indicates the presence of spatial autocorrelation.

Hot spot analysis (Getis-OrdGi* statistic)

Getis-OrdGi* statistics were computed to measure how spatial autocorrelation varies over the study location by calculating GI* statistic for each area. Z-score is computed to determine the statistical significance of clustering, and the p-value computed for the significance. Statistical output with high GI* indicates "hotspot" whereas low GI* means a "cold spot"(32).

Spatial interpolation

It is very expensive and laborious to collect reliable data in all areas of the country to know the burden of a certain event. Therefore, part of a certain area can be predicted by using observed data using a method called interpolation. The spatial interpolation technique is used to predict abortion on the un-sampled areas in the country based on sampled EAs. There are various deterministic and geostatistical interpolation methods. Among all of the methods, ordinary Kriging and empirical Bayesian Kriging are considered the best method since it incorporates the spatial autocorrelation and it statistically optimizes the weight(33). Ordinary Kriging spatial

interpolation method was used for this study for predictions of abortion in unobserved areas of Ethiopia.

Spatial scan statistical analysis

Spatial scan statistical analysis Bernoulli based model was employed to test for the presence of statistically significant spatial clusters of abortion using Kuldorff's SaTScan version 9.6 software. The spatial scan statistic uses a circular scanning window that moves across the study area. Women having pregnancy termination were taken as cases and those who have not as controls to fit the Bernoulli model. The numbers of cases in each location had Bernoulli distribution and the model required data for cases, controls, and geographic coordinates. The default maximum spatial cluster size of <50% of the population was used, as an upper limit, which allowed both small and large clusters to be detected and ignored clusters that contained more than the maximum limit.

For each potential cluster, a likelihood ratio test statistic and the p-value was used to determine if the number of observed abortion within the potential cluster was significantly higher than expected or not. The scanning window with maximum likelihood was the most likely performing cluster, and the p-value was assigned to each cluster using Monte Carlo hypothesis testing by comparing the rank of the maximum likelihood from the real data with the maximum likelihood from the random datasets. The primary and secondary clusters were identified and assigned p-values and ranked based on their likelihood ratio test, based on 999 Monte Carlo replications(34).

Ethics approval and consent to participate

Permission to get access to the data was obtained from the measure DHS program online request from http://www.dhsprogram.com.website and the data used were publicly available with no personal identifier.

Result

Socio-demographic characteristics of respondents

A total of 15,683 women were included in the 2016 EDHS survey. Of these, a weighted sample of 12,378 women was included in this study. Of these, 89% (N=11,048) were rural residents and 44.1% (N=5,457) lived in Oromia region. Regarding educational status, 66.8% (N=8,267) stated they had no formal education. The median age of women was 29 years (IQR± 9 years). In terms of marital status, 93.7% (N=11,595) of respondents were married (Table 1).

Obstetric and socioeconomic characteristics of respondents

Less than half 44.4% (N=5,495) of the respondents had four and above birth and 23.9% (N=2,969) of women were from the poorest household. Concerning listening to the radio, about 73.6% (N=9,111) of respondents never listened to the radio (Table 2).

The prevalence of abortion among women in Ethiopia, 2016

The result revealed that about 8.9% (N=1,105) [95% CI (8.4%-9.5%)] of the respondents in Ethiopia reported had pregnancy termination. The study reported that about 9.2% (N=1,016) of rural residents and 6.7% (N=89) of urban residents ever had a pregnancy terminated. The prevalence of abortion varies across regions ranging from 4.5% (N=6) in Benishangul-gumuz to 11.3% (N=89) in the Tigray region (Figure 2).

Spatial distribution of abortion

Each point on the map represents one census enumeration area which encompasses several abortion cases. The red color indicates areas with a high prevalence of abortion, whereas the green one indicates a low prevalence of abortion. In this study, the high prevalence of abortion was found in the Central and Northern Tigray, West Afar, East Benishangul-Gumuz, and East Southern Nation Nationalities and peoples (SNNPRs). High prevalence of abortion found in the Gambella, West Benishangul-Gumuz, central Oromia, Harari and Dire Dawa (Figure 3).
The result showed that the spatial distribution of abortion among reproductive-age women was nonrandom with Global Moran's Index that the spatial distribution of ARI among under-five

children was nonrandom with Global Moran's I 0.06 ($p<0.001$). The clustered patterns (on the right sides) showed that high rates of abortion occurred across the country. A z-score 3.54 indicated that there was less than 1% likelihood for this clustered pattern to be the result of a random chance. The bright red and blue colors at the end tails indicated an increased significance level (Figure 4).

Hotspot analysis of abortion

The hotspot analysis using Getis-OrdGi* statistic revealed that significant hot spot areas (area with high abortion rate) were identified in most parts of Tigray, Addis Ababa, South Amhara and West SNNPRs regions whereas significant cold spot areas were detected in most parts of Gambella and Benishangul-Gumuz regions (Figure 5).

Kriging Interpolation of Abortion

Based on EDHS 2016 sampled data, the Kriging interpolation technique predict that the highest abortion rate was detected in Northern Tigray, Addis Ababa, Southwest Oromia, Southwest SNNPRs and Northern Afar. In contrast, the relatively low abortion rate was detected in Gambella, South Amhara, and West Benishangul-Gumuz and the Eastern part of Afar regions (Figure 6).

Spatial Scan Statistical analysis

A total of 60 significant clusters within 4 spatial windows were identified at which 19 clusters were primary (most likely clusters). The primary clusters were located in the Northern part of the Tigray region. Which were centered at 14.175601 N, 38.891649 E with 62.42 km radius, a Relative Risk (RR) of 2.63, and Log-Likelihood Ratio (LRR) of 26.6, at p-value<0.01. It showed that women within the spatial window had 2.63 times higher risk for abortion as compared to women outside the spatial window (Table 3). Whereas, the secondary clusters were located in border areas between Oromia and Amhara, southeast Oromia and border areas between SNNPRs and Oromia regions. The bright red color circular window (Rings) indicates that the most statistically significant spatial window contains primary clusters of abortion (Figure 7). There was a higher risk of abortion within the window than outside the window.

Determinants of abortion

Model comparison: AIC, BIC, and Deviance were checked, and the mixed effect logistic regression model was chosen because of the smallest value of Deviance since the model was nested model (Table 4). Furthermore, the ICC value was 0.21(0.17, 0.26) and the Log-likelihood ratio test was (chibar2 (01) = 238.49 Prob >= chibar2 = 0.0000) which informed us to choose Mixed effect logistic regression model (GLMM) over the basic model.

In the mixed-effect logistic regression model, maternal age, religion, residence, wealth status, educational status, frequency of watching television, frequency of listening radio, birth history, and marital status showed a significant association in the bi-variable analysis and were considered for multivariable analysis.

However, in the multivariable mixed-effect logistic regression analysis, educational status, maternal age, frequency of watching television, frequency of listening radio, and religion have remained statistically significant.

The odds of experiencing abortion were nearly 5 times [AOR=4.96, 95%CI (3.42, 7.18)] in rural resident women as compared to urban women. Protestant religious followers had 44 % [AOR=0.56, 95%CI (0.42, 0.75)] decreased odds of having an abortion as compared to orthodox Christians.

The odds of experiencing abortion were 2.2 [AOR=2.20; 95%CI(1.27, 3.80)], [AOR=3.2; 95%CI(1.82, 5.71)], [AOR=3.01; 95%CI(1.67, 5.42)], [AOR=4.57;95%CI(2.47, 8.46)], [AOR=3.12; 95%CI(1.52, 6.44)] times higher among women in the age group 24-29, 30-34, 35-39, 40-44 and 45 -49 years as compared to women 15-19 years old. The likelihood of experiencing abortion among women with primary education was 1.36 times higher than among women with no education [AOR=1.36; 95%CI (1.13, 1.64)]. Women with the richest wealth index were 1.72 times higher chance of abortion as compared to women with the poorest wealth status [AOR=1.72; 95% CI(1.24, 2.40)].

The odds of having abortion among women who listened to the radio less than once a week were 1.27 times higher than women who never listened to the radio (AOR=1.27, CI: 1.01, 1.60). Women who watched television at least once a week were 1.45 times more likely to had an

abortion as compared to women who never watched the television [AOR=1.45 95%CI (1.04, 2.01)] (Table 5).

Discussion

Exploring the spatial distribution of abortion is crucial to identify aggregations of abortion cases to target public health interventions.

The current investigation revealed that the prevalence of abortion in Ethiopia was 8.9 % with significant spatial variability. The finding indicates that abortion is a public health problem in Ethiopia (35). The spatial scan statistics detected a total of four statistically significant SaTScan clusters of areas with a high prevalence of abortion. In multivariable mixed-effect logistic regression analysis, wealth status, maternal education, religion, media exposure, and maternal age were significantly associated with experiencing abortion.

The current finding was consistent with national based study in Mozambique (9.0%) (36) but lower than the national level study in and Ghana (25%) (36) and northwest Ethiopia (19%) (37). This might be attributed to the difference in the study period and improvement in the quality of data to date. It was found to be higher than those of studies done in India (1.7%) (38) and Wolayita-sodo, Ethiopia (6.5%) (39). The difference might be due to the difference in the study population. The current investigation was conducted at the national level (community-based) based on EDHS 2016 which is supposed nationally representative while the study in Wolayita-sodo was conducted among university students (facility-based).

This study demonstrated that abortion had statistically significant geographic variation which was non-randomly distributed. From the 2016 EDHS data SaTScan analysis, the most likely primary SaTScan clusters (hotspot) areas were detected in the northern part of the Tigray region whereas the secondary clusters were detected in border areas of Oromia, Amhara, and SNNP. The spatial variation might be related to the difference in socioeconomic status and health inequality within the country. There is a gap in health service utilization like family planning, ANC and other reproductive health services across regions (40).

The mixed-effect logistic regression analysis revealed that terminating pregnancy among women was significantly associated with the place of residence. Women residing in rural areas were

more likely to experience abortion as compared to urban residents. This finding was consistent with a study done in northwest Ethiopia (37) and India (38). This might be due to lack of access to abortion services, and limited information about abortion due to lack of access to media in the urban areas (41).

Maternal age was found to be significantly associated with experiencing abortion. Women in the age group 25-29, 30-34, 35-39, 40-44, and 45-49 years were more likely to experience abortion than women in the age group 15-19. This was consistent with findings in Ghana (36), Denmark (42) and Mozambique (36). The possible explanation could be that older women are more likely to have medical and pregnancy-related complications like high blood pressure (HTN), Diabetic Mellitus (DM), cardiovascular diseases and chromosomal abnormality that can complicate the pregnancy and increase the risk of poor pregnancy outcome (43). When these complications are not well controlled, it can contribute to miscarriage, intra uterine growth restriction (IUGR) and birth defects such as neural tube defect, Down syndrome, anencephaly and as maternal age increases the risk of chromosomal abnormality increased, decreased uterine and hormonal function (44).

We found experiencing abortion was importantly related to access to media exposure (televisions and radio) as compared to women who had no access to media. This result agrees with reports in Ghana and Mozambique (36). The possible reason might be that since media is an important mechanism in providing information about how and where to terminate a pregnancy. Furthermore, women who have media exposure might be aware of available laws related to abortion and less likely to be stigmatized by society (45).

The study also showed that the likelihood of experiencing abortion among protestant religious followers was lower as compared to those women who are orthodox Christians. This was consistent with a study in China (46). This might be due to lack of access to reproductive health services like abortion services, lack of awareness, cultural and religious belief since orthodox religious followers had deep-rooted belief towards abortion they belief as sinning (46). Regarding wealth status, the richest women had higher odds of experiencing abortion as compared to the poorest women. This was similar to a study in Ghana (47) and Nepal (48). The probable reason might be that the wealth status of women can determine their ability to cover the cost of health care services and also poor women faced cost barriers like transportation costs

since the abortion services did not perform elsewhere, this can impede women to have an abortion.

Maternal education was a significant predictor of experiencing abortion. It showed that women with primary education were highly likely to experience abortion as compared to women with no education. This was consistent prior study conducted in northwest Ethiopia(37), and India (38), could be due to the reason that educated women didn't need to give birth to meet the demands of ongoing education, to postpone birth interval in order to improve pregnancy outcome and the educated women had information and access to the abortion services. The strength of this study was that it was based on weighted large, nationally representative data. The study is done using an advanced model to take into account the clustering effect (mixed-effect logistic regression) to get reliable standard error and estimate. However, the study finding is interpreted in light of limitations. First, as with other cross-sectional studies, the temporal relationship can't be established. Second, since data was collected from self-report from respondents there may be a possibility of social desirability bias since abortion is a sensitive issue can lead to under ported.

Conclusions

The finding of this study helps to fill the gap in the spatial distribution and determinant factors with abortion at the national level. The study showed that there was significant spatial variation within Ethiopia and maternal education, wealth status, media exposure, and religion were significant factors associated with abortion. We recommend that the health sectors, NGOs and the government strengthen the effort towards reproductive health services like family planning services particularly for rural residents and should design effective public health interventions in the identified hotspot areas to reduce the incidence of abortion and abortion-related mortality. Besides, we recommend scholars to examine the reason that abortion had significant geographic variation within the countries using a detail exploration like qualitative study.

Abbreviations

AIC=Akakie information criteria; AOR=adjusted odds ratio; AOR=Adjusted odds ratio; BIC=Bayesian information criterion; CI=confidence interval; COR=crude odds ratio; DHS=Demographic health survey; DM=diabetic mellitus; EAs=Enumeration areas;

EDHS=Ethiopian demographic and health survey; GLMM=Generalized linear mixed model; HTN=Hypertension; ICC=Intra cluster correlation coefficient; IUGR=Intra uterine growth restriction; LLR=Log likelihood ratio; LR=Likelihood ratio; RR=Relative risk; SNNP=Southern nations and nationalities of people; SSA=Sub-Saharan Africa

Availability of data and materials

Data is available online and you can access it from www.measuredhs.com.

Competing Interests

Authors declare that they have no conflict of interest

Funding

No funding was obtained for this study.

Authors' contribution

Conceptualization: Getayeneh Antehunegn Tesema, Tesfaye Hambisa Mekonnen, Achamyeleh Birhanu Teshale

Data curation: Getayeneh Antehunegn Tesema, Tesfaye Hambisa Mekonnen, Achamyeleh Birhanu Teshale

Investigation: Getayeneh Antehunegn Tesema, Tesfaye Hambisa Mekonnen, Achamyeleh Birhanu Teshale

Methodology: Getayeneh Antehunegn Tesema, Tesfaye Hambisa Mekonnen, Achamyeleh Birhanu Teshale

Software: Getayeneh Antehunegn Tesema, Tesfaye Hambisa Mekonnen, Achamyeleh Birhanu Teshale

Validation: Getayeneh Antehunegn Tesema, Tesfaye Hambisa Mekonnen, Achamyeleh Birhanu Teshale

Writing: Getayeneh Antehunegn Tesema, Tesfaye Hambisa Mekonnen, Achamyeleh Birhanu Teshale

Writing – review and editing: Getayeneh Antehunegn Tesema, Tesfaye Hambisa Mekonnen, Achamyeleh Birhanu Teshale

Consent for publication

Not applicable

Acknowledgments

We would like to thank the measure DHS program for providing the data set.

References

1. Singh S, Maddow-Zimet I. Facility-based treatment for medical complications resulting from unsafe pregnancy termination in the developing world, 2012: a review of evidence from 26 countries. BJOG: An International Journal of Obstetrics & Gynaecology. 2016;123(9):1489-98.
2. Say L, Chou D, Gemmill A, Tunçalp Ö, Moller A-B, Daniels J, et al. Global causes of maternal death: a WHO systematic analysis. The Lancet Global Health. 2014;2(6):e323-e33.
3. Ronsmans C, Graham WJ, group LMSSs. Maternal mortality: who, when, where, and why. The lancet. 2006;368(9542):1189-200.
4. Singh S, Remez L, Sedgh G, Kwok L, Onda T. Abortion worldwide 2017: uneven Progress and unequal AccessAbortion worldwide 2017: uneven Progress and unequal Access. 2018.
5. Khan KS, Wojdyla D, Say L, Gülmezoglu AM, Van Look PF. WHO analysis of causes of maternal death: a systematic review. The lancet. 2006;367(9516):1066-74.
6. Singh S, Fetters T, Gebreselassie H, Abdella A, Gebrehiwot Y, Kumbi S, et al. The estimated incidence of induced abortion in Ethiopia, 2008. International perspectives on sexual and reproductive health. 2010:16-25.
7. Singh S, editor The global magnitude and consequences of unsafe abortion. Gutmacher Institute Regional meeting on Post-abortion Care Alexandria, Egypt; 2010.
8. Olukoya A, Kaya A, Ferguson B, AbouZahr C. Unsafe abortion in adolescents. International Journal of Gynecology & Obstetrics. 2001;75(2):137-47.
9. Klima CS. Unintended pregnancy: consequences and solutions for a worldwide problem. Journal of Nurse-Midwifery. 1998;43(6):483-91.
10. Sedgh G, Bearak J, Singh S, Bankole A, Popinchalk A, Ganatra B, et al. Abortion incidence between 1990 and 2014: global, regional, and subregional levels and trends. The Lancet. 2016;388(10041):258-67.
11. Grimes DA, Benson J, Singh S, Romero M, Ganatra B, Okonofua FE, et al. Unsafe abortion: the preventable pandemic. The lancet. 2006;368(9550):1908-19.
12. Sedgh G, Singh S, Shah IH, Åhman E, Henshaw SK, Bankole A. Induced abortion: incidence and trends worldwide from 1995 to 2008. The Lancet. 2012;379(9816):625-32.

13. Henshaw SK. Induced abortion: a worldwide perspective. Family Planning Perspectives. 1986;18(6):250-4.

14. Okonofua FE, Onwudiegwu U, Odunsi O. Illegal induced abortion: a study of 74 cases in Ile-Ife, Nigeria. Tropical Doctor. 1992;22(2):75-8.

15. Dixon-Mueller R. Abortion policy and women's health in developing countries. International Journal of Health Services. 1990;20(2):297-314.

16. Singh K, Ratnam S. The influence of abortion legislation on maternal mortality. International Journal of Gynecology & Obstetrics. 1998;63:S123-S9.

17. 2016. CSACEaI. Ethiopia Demographic and Health Survey 2016. Addis Ababa, Ethiopia, and Rockville, Maryland, USA: CSA and ICF. 2016.

18. Tesfaye G, Hambisa MT, Semahegn A. Induced abortion and associated factors in health facilities of Guraghe zone, southern Ethiopia. Journal of pregnancy. 2014;2014.

19. Powell-Griner E, Trent K. Sociodemographic determinants of abortion in the United States. Demography. 1987;24(4):553-61.

20. Misago C, Fonseca W, Correia L, Fernandes LM, Campbell O. Determinants of abortion among women admitted to hospitals in Fortaleza, North Eastern Brazil. International journal of epidemiology. 1998;27(5):833-9.

21. Bose S, Trent K. Socio-demographic determinants of abortion in India: a north–south comparison. Journal of biosocial science. 2006;38(2):261-82.

22. Filippi V, Ronsmans C, Campbell OM, Graham WJ, Mills A, Borghi J, et al. Maternal health in poor countries: the broader context and a call for action. The Lancet. 2006;368(9546):1535-41.

23. Samandari G, Wolf M, Basnett I, Hyman A, Andersen K. Implementation of legal abortion in Nepal: a model for rapid scale-up of high-quality care. Reproductive health. 2012;9(1):7.

24. Parkhurst JO, Penn-Kekana L, Blaauw D, Balabanova D, Danishevski K, Rahman SA, et al. Health systems factors influencing maternal health services: a four-country comparison. Health policy. 2005;73(2):127-38.

25. Martins-Melo FR, Lima MdS, Alencar CH, Ramos Jr AN, Carvalho FHC, Machado MMT, et al. Temporal trends and spatial distribution of unsafe abortion in Brazil, 1996-2012. Revista de saude publica. 2014;48:508-20.

26. Ba-Thike K. Abortion: a public health problem in Myanmar. Reproductive Health Matters. 1997;5(9):94-100.

27. Organization WH. Evaluating the quality of care for severe pregnancy complications: the WHO near-miss approach for maternal health. 2011.

28. Central statistical agency(CSA) I. Ethiopian Demographic and Health survey. Addis Abeba: Addis Ababa, Ethiopia, and Rockville, Maryland, USA: CSA and ICF, 2016.

29. Adugna A. Health Institutions and Services. Addis Abeba: July 2014.

30. ICF CSACEa. Ethiopia Demographic and Health Survey 2016 Addis Ababa, Ethiopia, and Rockville , maryland, USA: CSA and ICF. 2016.

31. Waldhör T. The spatial autocorrelation coefficient Moran's I under heteroscedasticity. Statistics in Medicine. 1996;15(7-9):887-92.

32. Tsai P-J, Lin M-L, Chu C-M, Perng C-H. Spatial autocorrelation analysis of health care hotspots in Taiwan in 2006. BMC Public Health. 2009;9(1):464.

33. Bhunia GS, Shit PK, Maiti R. Comparison of GIS-based interpolation methods for spatial distribution of soil organic carbon (SOC). Journal of the Saudi Society of Agricultural Sciences. 2018;17(2):114-26.

34. Kulldorff M. SaTScanTM user guide. Boston; 2006.

35. Abdella A. Maternal mortality trend in Ethiopia. Ethiopian Journal of Health Development. 2010;24(1).

36. Dickson KS, Adde KS, Ahinkorah BO. Socio–economic determinants of abortion among women in Mozambique and Ghana: evidence from demographic and health survey. Archives of Public Health. 2018;76(1):37.

37. Senbeto E, Alene GD, Abesno N, Yeneneh H. Prevalence and associated risk factoprs of Induced Abortion in Northwet Ethiopia. Ethiopian Journal of health development. 2005;19(1):37-44.

38. Pallikadavath S, Stones RW. Maternal and social factors associated with abortion in India: a population-based study. International Family Planning Perspectives. 2006:120-5.

39. Gelaye AA, Taye KN, Mekonen T. Magnitude and risk factors of abortion among regular female students in Wolaita Sodo University, Ethiopia. BMC women's health. 2014;14(1):50.

40. Kongsri S, Limwattananon S, Sirilak S, Prakongsai P, Tangcharoensathien V. Equity of access to and utilization of reproductive health services in Thailand: national Reproductive Health Survey data, 2006 and 2009. Reproductive Health Matters. 2011;19(37):86-97.

41. Pearson TA, Lewis C. Rural epidemiology: insights from a rural population laboratory. American journal of epidemiology. 1998;148(10):949-57.

42. Andersen A-MN, Wohlfahrt J, Christens P, Olsen J, Melbye M. Maternal age and fetal loss: population based register linkage study. Bmj. 2000;320(7251):1708-12.

43. Mannino DM, Thorn D, Swensen A, Holguin F. Prevalence and outcomes of diabetes, hypertension and cardiovascular disease in COPD. European Respiratory Journal. 2008;32(4):962-9.

44. Assessment PR. Down syndrome: prenatal risk assessment and diagnosis. Am Fam Physician. 2000;62(4):825-32.

45. Cook RJ, Dickens BM. Reducing stigma in reproductive health. International Journal of Gynecology & Obstetrics. 2014;125(1):89-92.

46. Pinter B, Hakim M, Seidman DS, Kubba A, Kishen M, Di Carlo C. Religion and family planning. The European Journal of Contraception & Reproductive Health Care. 2016;21(6):486-95.

47. Sundaram A, Juarez F, Bankole A, Singh S. Factors associated with abortion-seeking and obtaining a safe abortion in Ghana. Studies in Family Planning. 2012;43(4):273-86.

48. Mehata S, Menzel J, Bhattarai N, Sharma SK, Shah M, Pearson E, et al. Factors associated with induced abortion in Nepal: data from a nationally representative population-based cross-sectional survey. Reproductive health. 2019;16(1):68.

Figure legends

Figure 1: Map of the study area (source: CSA, 2013)

Figure 2: The regional prevalence of abortion among reproductive age women in Ethiopia, 2016 (source: CSA, 2013)

Figure 3: The spatial distribution of abortion in Ethiopia, 2016 (source: CSA, 2013)

Figure 4: The Global spatial autocorrelation of abortion in Ethiopia, 2016 (source: CSA, 2013)

Figure 5: The Hotspot analysis of abortion in Ethiopia, 2016 (source: CSA, 2013)

Figure 6: The Kriging interpolation of abortion in Ethiopia, 2016 (source: CSA, 2013)

Figure 7: The SaTScan analysis of abortion in Ethiopia, 2016 (source: CSA, 2013)

Table 1: Socio-demographic characteristics of respondents in Ethiopia, 2016 (N=12,378).

Variables	Weighted frequency(N)	Percent (%)
**Residence**		
Urban	1,330	11.0
Rural	11,048	89.0
**Region**		
Tigray	790	6.4
Afar	131	1.1
Amhara	2,300	18.6
Oromia	5,457	44.1
Somali	587	4.7
Ben-Gumuz	138	1.1
Gambella	2,601	21.0
Harari	29	0.2
Addis Ababa	262	2.1
Dire Dawa	52	0.4
**Maternal age (in years)**		
15-19	380	3.0
20-24	2,223	18.0
25-29	3,736	30.2
30-34	2,872	23.2
35-39	2,002	16.2
40-44	873	7.0
45-49	293	2.4
**Maternal educational status**		
No education	8,267	66.8
Primary	3,261	26.3
Secondary	555	4.5
Higher	296	2.4
**Religion**		

Orthodox	4,210	34.0
Muslim	5,102	41.2
Catholic	113	0.9
Protestant	21.5	21.5
Others*	293	2.4
**Husband education**		
No education	5,682	45.9
Primary	4,572	36.9
Secondary	868	7.0
Higher	1,257	10.2
**Marital status**		
Never married	65	0.5
Married	11,595	93.7
Living with partner	137	1.1
Widowed	145	1.2
Divorced	311	2.5
Separated	127	1.0

Keys: *= Traditional religious followers

Table 2: Obstetric and socio-economic characteristics of participants in Ethiopia, 2016.

Characteristics (N=12,378)	Weighted frequency (N)	Percentage (%)
**Wealth status**		
Poorest	2,969	23.9
Poor	2,799	22.6
Middle	2,565	20.7
Richer	2,275	18.4
Richest	1,770	14.3
**Frequency of listening the radio**		
Not at all	9,111	73.6
Less than once a week	1,624	13.2
At least once a week	1,643	13.3
**Frequency of watching the television**		
Not at all	10,163	82.1
least than once a week	1,233	10.0
At least once a week	983	7.9
**Occupational status**		
Unemployed	8,740	70.6
Employed	3,638	29.4
**Birth history**		
No birth	1,479	12.0
One birth	1,893	15.3
Two births	1,869	15.1
Three births	1,642	13.2
Four and above births	5,495	44.4
**Preceding birth interval**		
Less than 24 month	2,353	23.4
≥ 24 months	7,706	76.6
**Terminated pregnancy (abortion)**		

No	11,273	91.1
Yes	1,103	8.9
Smoking status		
Yes	12,285	99.2
No	93	0.8

Table 3: Significant spatial clusters of abortion among women in Ethiopia, 2016.

clusters	Enumeration areas(EAs)/ clusters detected	Coordinates/radius	Population	Cases	RR	LLR	P-value
1	84, 45, 81, 590, 481, 461, 400, 636, 597, 89, 479, 604, 156, 355, 598, 584, 404, 226, 579	(14.175601 N, 38.891649 E) / 62.42 km	327	70	2.63	26.6	<0.001
2	452, 472, 286, 289, 123	(7.410925 N, 40.475707 E) / 85.79 km	125	27	2.58	10.2	0.01
3	92	(6.708449 N, 44.273542 E) / 0 km	34	12	4.19	9.5	0.03
4	510, 267, 572, 10, 423, 350, 229, 482, 460, 206, 176, 531, 218, 310,617, 120, 637, 517, 112, 201, 274, 463, 144, 464, 532, 91, 369, 170, 11, 153, 287, 339, 626, 107, 247	(10.160658 N, 38.634847 E) / 125.60 km	412	61	1.79	9.2	0.04
5	50, 342, 86, 21, 503, 450, 574, 182, 505, 398	(5.546952 N, 37.666334 E) / 88.77 km	267	42	1.89	7.5	0.171
6	276	(10.717422 N, 40.344525 E) / 0 km	25	9	4.26	7.3	0.218
7	564, 39, 230, 51	(9.555410 N, 40.326165 E) / 34.04 km	61	15	2.92	7.08	0.245

Table 4: Model comparison between standard logistic regression and mixed effect logistic regression.

Model comparison	AIC	BIC	Deviance
Logistic regression model	6856.17	7077.95	6796.09
Mixed effect logistic regression model	6622.02	6851.19	6560.02

Table 5; Bi-variable and Multivariable mixed effect logistic regression analysis of determinants with Abortion among reproductive age women in Ethiopia from January 18 to June 27, 2016.

Variable	Terminated pregnancy		COR (95% CI)	AOR (95% CI)
	No	Yes		
**Residence**				
Urban	1,983	130	1	1
Rural	8,989	897	2.28 (1.70, 3.08)	4.96 (3.42, 7.18)**
**Age**				
15-19	390	18	1	1
20-24	2,211	124	1.17 (0.69, 1.98)	1.27 (0.74, 2.19)
25-29	3,280	282	1.79 (1.08, 2.97)	2.20 (1.27, 3.80)**
30-34	2,443	279	2.51 (1.51, 4.16)	3.23 (1.82, 5.71)**
35-39	1,758	192	2.29 (1.37, 3.82)	3.01 (1.67, 5.42)**
40-44	670	107	3.51 (2.05, 6.00)	4.57 (2.47, 8.46)**
45-49	220	25	2.36 (1.22, 4.55)	3.12 (1.52, 6.44)**
**Wealth status**				
Poorest	4,166	387	1	1
Poorer	1,848	149	0.89 (0.71, 1.12)	0.85 (0.67, 1.07)
Middle	1,490	154	1.16 (0.92, 1.46)	1.07 (0.84, 1.36)
Richer	1,361	128	1.01 (0.79, 1.30)	0.91 (0.70, 1.19)
Richest	2,107	209	1.14 (0.90, 1.44)	1.72 (1.24, 2.40)*
**Educational status**				
No education	7,158	670	1	1
Primary	2,688	269	1.09 (0.92, 1.29)	1.36 (1.13, 1.64)**
Secondary	740	55	0.76 (0.55, 1.04)	0.98 (0.68, 1.41)
Higher	386	33	0.88 (0.58, 1.33)	0.99(0.62, 1.61)
**Religion**				

Orthodox	3,083	354	1	1
Muslim	5,647	518	0.75 (0.61, 0.93)	0.81 (0.64, 1.01)
catholic	75	3	0.38 (0.11, 1.31)	0.40 (0.12, 1.39)
Protestant	1,981	136	0.59 (0.45, 0.77)	0.56 (0.42, 0.75)**
Others	186	16	0.68 (0.36, 1.28)	0.66(0.34, 1.26)
**Frequency of listening radio**				
Not at all	8,456	733	1	1
Less than once a week	1,265	147	1.40 (1.14, 1.72)*	1.27 (1.01, 1.60)*
At least once a week	1,251	147	1.30 (1.05, 1.60)*	1.21 (0.96, 1.55)
**Frequency of watching television**				
Not at all	8,754	791	1	1
Less than once a week	877	102	1.35 (1.06, 1.73)	1.25 (0.95, 1.65)
At least once a week	1,341	134	1.19 (0.93, 1.51)	1.45 (1.04, 2.01)*
**Birth history**				
zero birth	1,416	96	1	1
One birth	1,822	142	1.16 (0.87, 1.53)	0.97 (0.72, 1.31)
Two births	1,649	146	1.31 (0.98, 1.73)	0.92(0.66, 1.27)
Three births	1,514	130	1.32 (0.99, 1.76)	0.85 (0.60, 1.19)
Four and above births	4,571	513	1.70 (1.33, 2.16)	0.85 (0.60, 1.19)
**Marital status**				
Married	10,191	967	1	1
Never married	273	28	1.11 (0.72, 1.69)	1.22 (0.78, 1.90)
Widowed	158	8	0.62 (0.29, 1.29)	0.52 (0.24, 1.11)
Divorced	349	24	0.74 (0.48, 1.15)	0.78 (0.49, 1.23)

Figure 1

Click here to download Figure Fig 1.tif

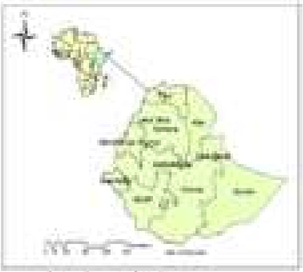

Figure 1 map of study area

Figure 2

Click here to download Figure Fig 2.tif

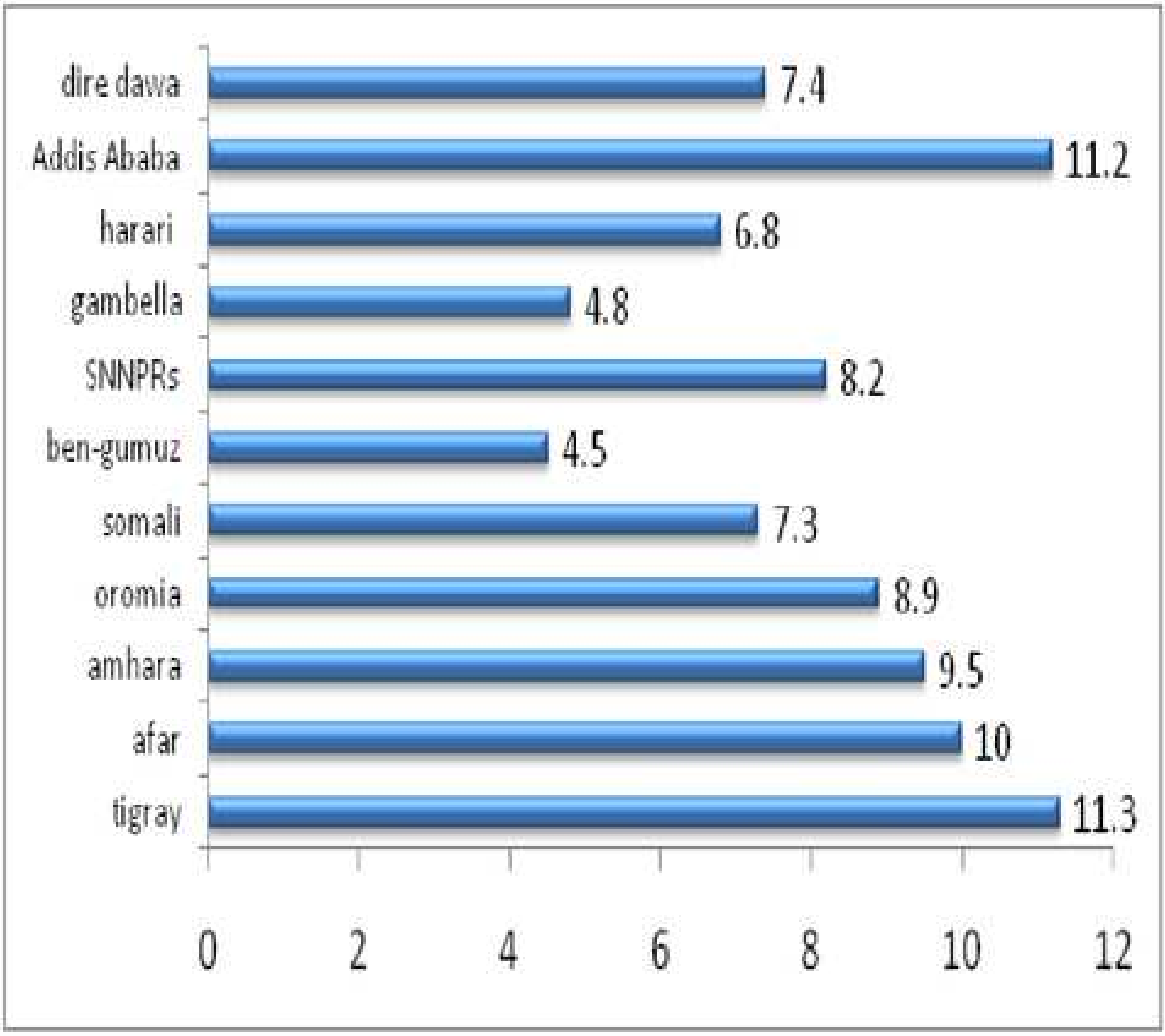

Figure 3 Click here to download Figure Fig 3.tif

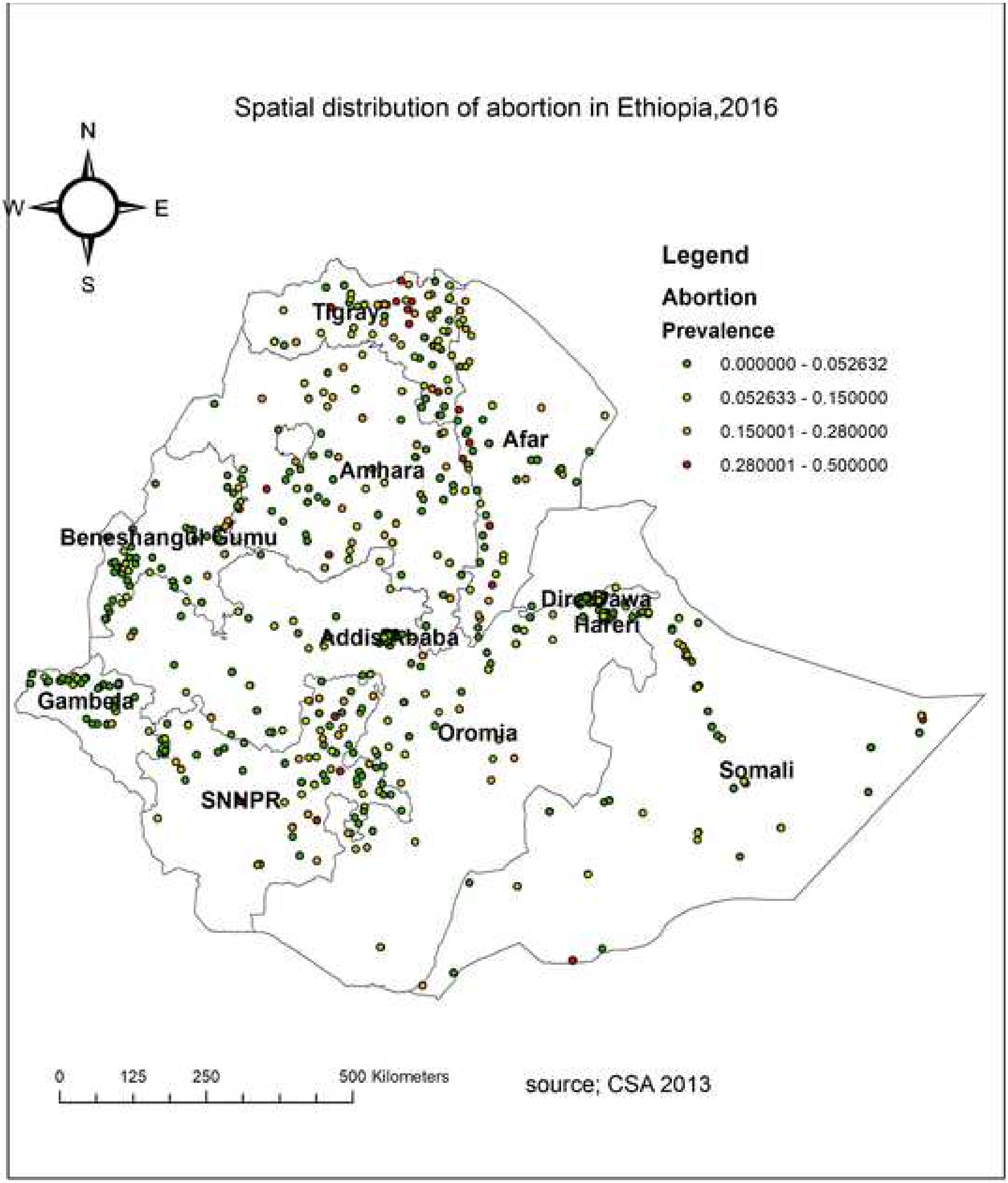

Figure 4

Click here to download Figure Fig 4.tif

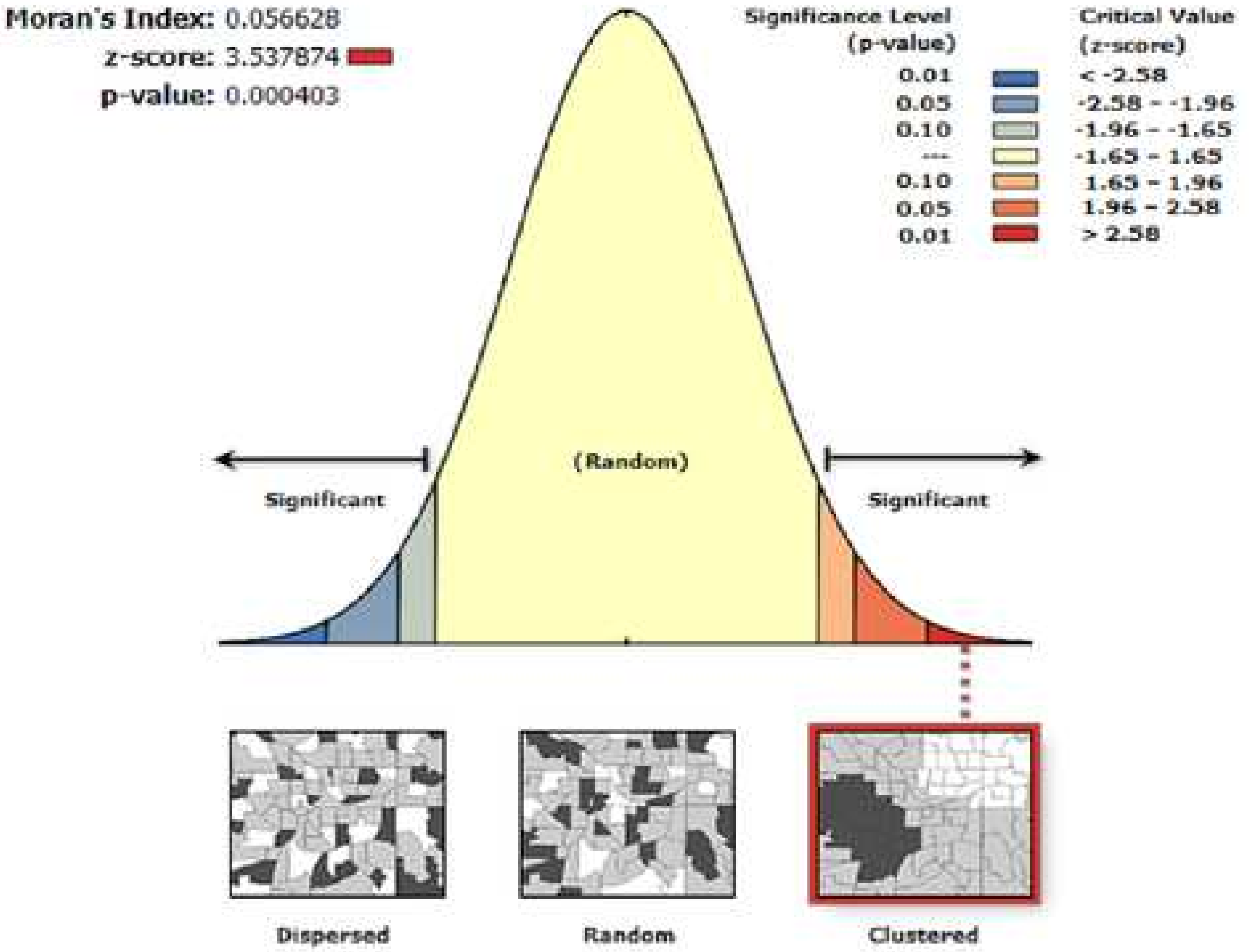

Given the z-score of 3.53787386448, there is a less than 1% likelihood that this clustered pattern could be the result of random chance.

Figure 5

Click here to download Figure Fig 5.tif

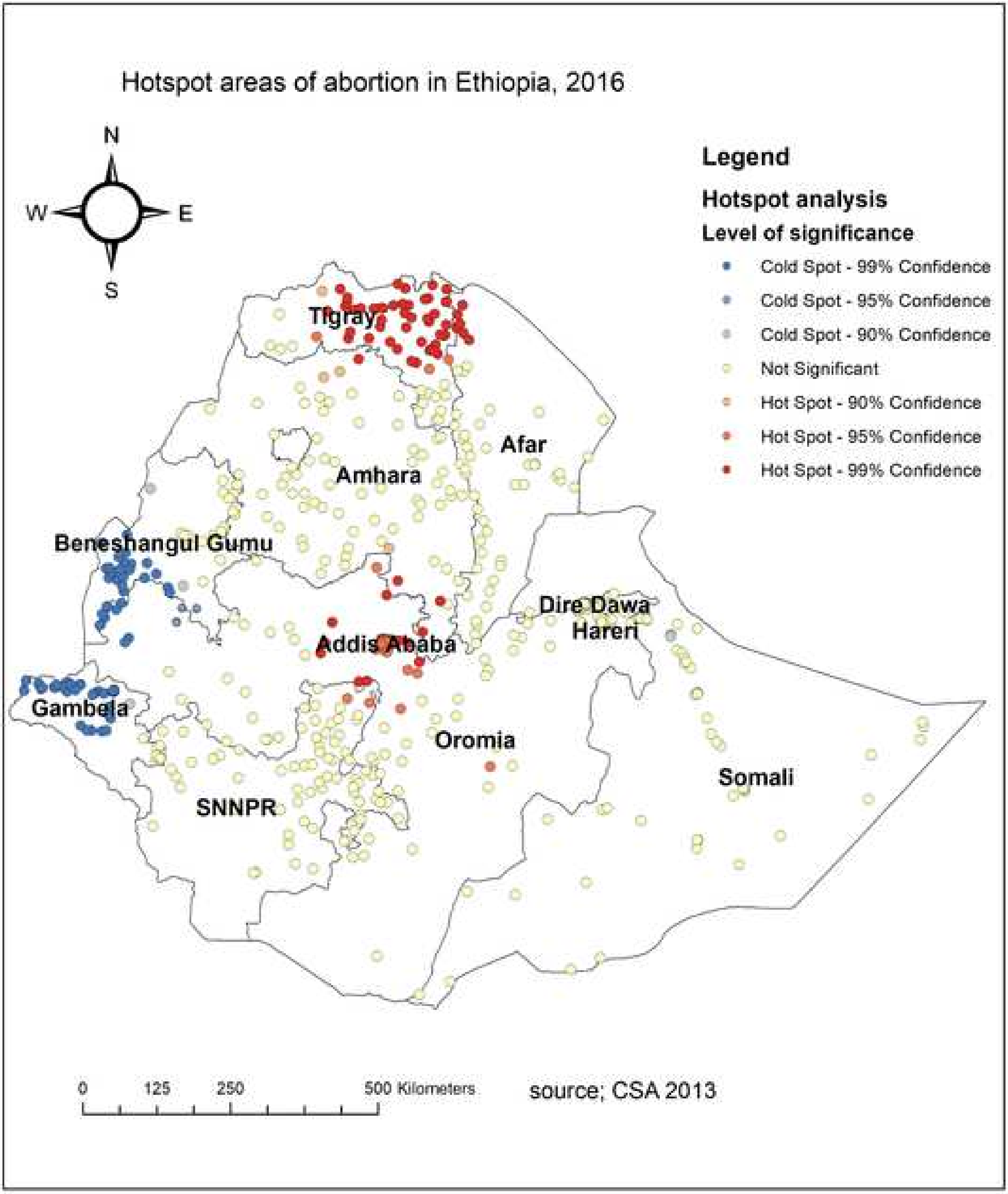

Figure 6

Click here to download Figure Fig 6.tif

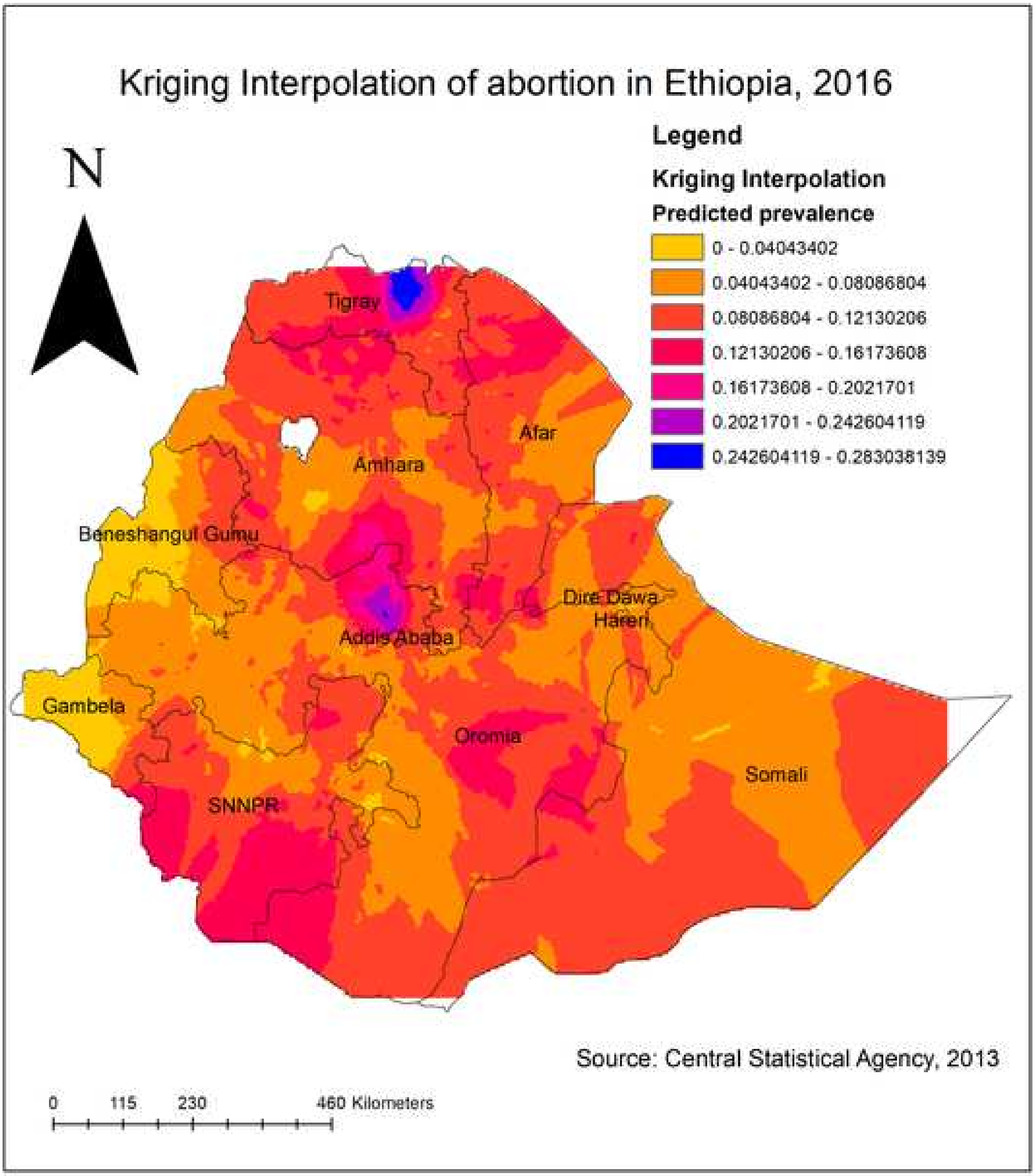

Figure 7

Click here to download Figure Fig 7.tif

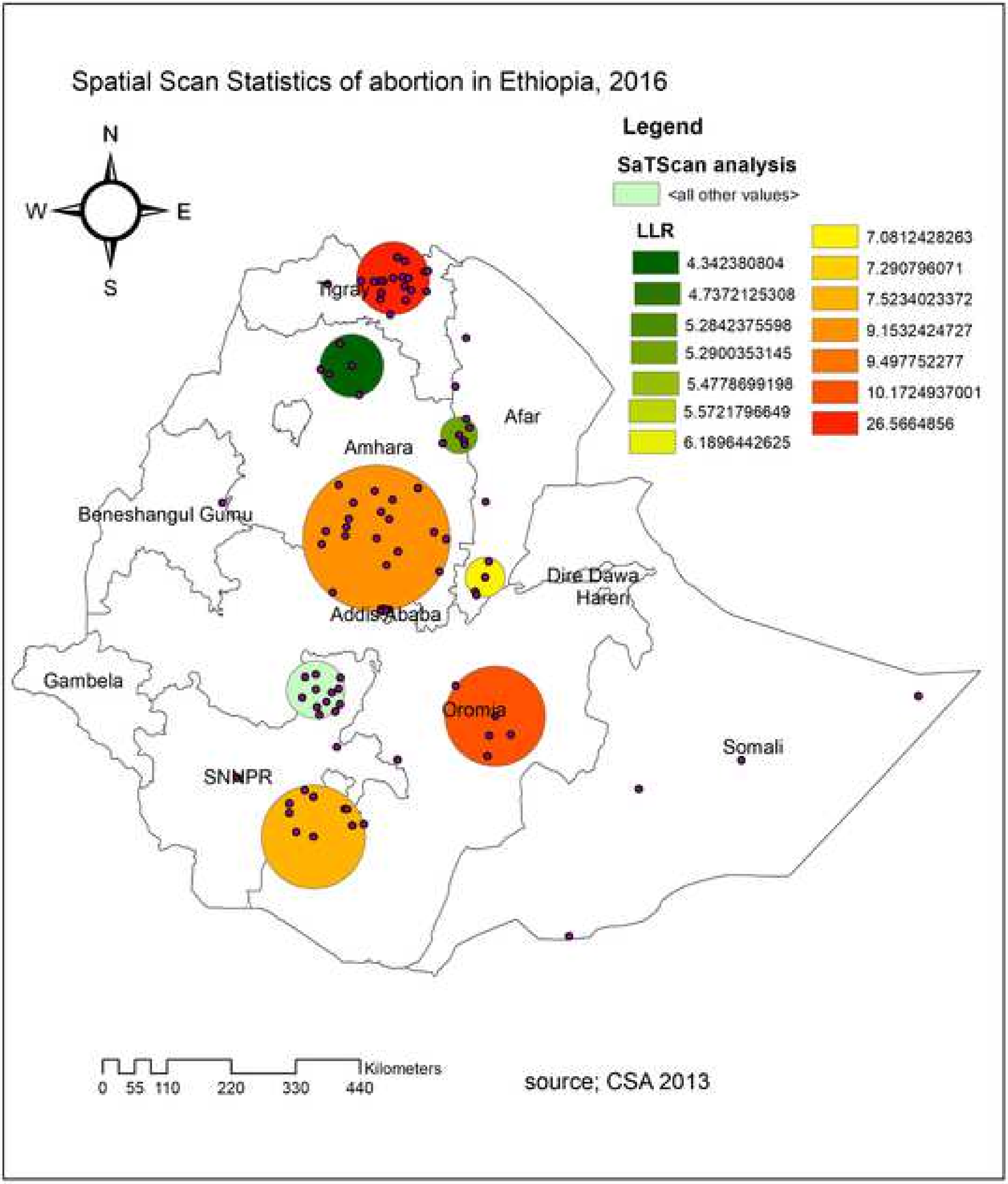

Supplement: Attachment — Submitted filename: Spatial distribution and determinants of abortion - Editors Comments.pdf [file pone.0235382.s001.pdf]
