# Supplementary material for: Spatial distribution and determinants of abortion among reproductive age women in Ethiopia, evidence from Ethiopian Demographic and Health Survey 2016 data: Spatial and mixed-effect analysis
Source: PLoS One. 2020 Jun 29;15(6):e0235382. doi: 10.1371/journal.pone.0235382 (PMC7323954; doi:10.1371/journal.pone.0235382)
Supplement: Attachment — Submitted filename: Point by Point response.docx [file pone.0235382.s002.docx]

**Point by point response for editors and reviewers comments**

**Manuscript title:** Spatial distribution and determinants of abortion among reproductive-age women in Ethiopia, Evidence from Ethiopian Demographic and Health Survey (EDHS) 2016 data: Spatial and Mixed-effect analysis

**Manuscript ID:** PONE-D-19-30532

**Dear editor/reviewer.**

Dear all,

We would like to thank you for these constructive, building and improvable comments on this manuscript that would improve the substance and content of the manuscript. We considered each comment and clarification questions of editors and reviewers on the manuscript thoroughly. Our point-by-point responses for each comment and questions are described in detail on the following pages. Further, the details of changes were shown by track changes in the supplementary document attached.

**Response to editors**

1. Please ensure that your manuscript meets PLOS ONE's style requirements, including those for file naming.
   **Authors’ response**: Thank you, editor. We have prepared the manuscript according to PLOS ONE’s style. (see the revised manuscript)
2. Thank you for stating the following financial disclosure: "N/A"

**Authors’ response:** Thank you, editor. As we have stated in the documents, the study was done based on Ethiopian Demographic and Health Survey (EDHS) which is already available at measure DHS program. We request this program by sending the objectives of the study and we receive an authorization letter from the DHS program.

3. PLOS requires an ORCID iD for the corresponding author in Editorial Manager on papers submitted after December 6th, 2016. Please ensure that you have an ORCID iD and that it is validated in Editorial Manager. To do this, go to ‘Update my Information’ (in the upper left-hand corner of the main menu), and click on the Fetch/Validate link next to the ORCID field. This will take you to the ORCID site and allow you to create a new iD or authenticate a pre-existing iD in Editorial Manager. Please see the following video for instructions on linking an ORCID iD to your Editorial Manager account: <https://www.youtube.com/watch?v=_xcclfuvtxQ>

**Authors’ response:** We have created ORCID

4. We note that Figures 1, 3 and 5-7 in your submission contain map images which may be copyrighted.

**Authors’ response:** Thank you, editors, for your concern. The map is not copyrighted rather we have done using GIS software based on the shapefile of Ethiopia received from Ethiopian Central Statistical Agency (CSA) by explaining the purpose of the study and GPS data (longitude and latitude) from measure DHS program by explaining the objective of the study through online requesting and allow us to access the shapefile and GPS data. Now we cite the source of the shapefile since it is needed to explore the spatial distribution of abortion.

**Response to reviewers’ comments**

**Reviewer # 1**

1. Statistics in the result section could be summarised better, eg not over repeating 95% CI, only present the most important and interesting results eg you don’t have to put all the statistics for all the age groups,

**Authors’ response**: Thank you, reviewer, for your valuable comment. We accepted and corrected it. (See the revised manuscript)

2. Some repetitions in the conclusions

**Authors’ response**: Thank you, reviewer. We rewrite the conclusion section and remove the repetitions. (See the revised manuscript)

- Line 88, what do you mean by the wide gap in abortions

**Authors’ response:** Thank you, reviewer. We have stated in the Introduction section as there is wide gap in abortions across and within countries meanwhile we reviewed kinds of literature conducted on abortion the prevalence of abortion varied across countries and within countries and this gives an insight that abortion has been varied across countries and spatial study is needed to identify which areas are significant hotspot.

- Revise some sentences, Line 113 missing full stop, Line 121, do you mean sample size

**Authors’ response:** Thank you, reviewer. We have corrected it. (See the revised manuscript)

- Statistics, I think one appropriate method of model evaluations and evaluations could be chosen and be used

**Authors’ response:** Thank you, reviewer. We used AIC, BIC, and Deviance for model comparison but mainly we depend on Deviance since our models are nested models and we used ICC, AIC, and BIC as supportive.

- Line 245-249, two sentences all talking about high prevalence, which regions do you refer to them as high prevalence

**Authors’ response:** Thank you, reviewer, for your comments. It was an editorial error and the second one was areas with a low prevalence of abortion, we had modified in the revised document. (see the revised document)

- Figures are of poor quality, can be improved to publication quality.

**Authors’ response:** Thank you, reviewer, we had improved the figure quality using PACE in TIFF format (see the revised document)

**Reviewer#2 #2:**
1. First, a thorough grammatical editing is necessary, as the very first sentence has a spelling error (uretro)

**Authors’ response:** Thank you, reviewer, for the valuable comments. We accepted the comments and we corrected the grammatical errors extensively the entire manuscript. For Uretro it is corrected as the uterus (see the revised manuscript)

2. Secondly, the figures are not helping your analysis. Figure 1 is virtually illegible, Figure 2 needs to be a map at the regional level, with correctly capitalized region/state names. Furthermore, Figure 3 needs help, the points are far too small for the reader to understand anything about the spatial variation in the rate, Figure 4 can be eliminated as the Moran I statistic can be reported in the text. Adjust the number of decimal places on the maps, there are too many decimal values, you should report at most 2 decimal places.

**Authors’ response:** Thank you, reviewer, for your valuable comments. We were included Figure 1 to show the study area on the map and as you said it is not that much important and we removed in the revised manuscript. We have modified Figure 2 in ascending order based on the prevalence of abortion across regions by capitalizing on the name of the region. For Figure 3 the points were placed in decimal places and know we put in terms of percentage of abortion at enumeration areas by reducing the decimal places. We had removed Figure 4 as it is well stated in the form of text. (See the revised manuscript)

- Regarding your statistical model, it's more common to model the spatial variation using a CAR random effect in the binomial model, and visualize the random effect or the smoothed rate map, versus doing separate kriging or scan statistic methods.

**Authors’ response:** Thank you, reviewer, for the comments. We have done Kriging interpolation analysis for predicting the prevalence of abortion in unsampled areas based on observed data and SaTScan analysis to identify hotspot areas of abortion by running circular windows but we haven't done the Conditional Autoregressive (CAR) model and visualize the random effect because there are no covariates collected at Enumeration Area (EAs) level in EDHS data. Since the GPS data were collected at EA level but the covariates were collected at the individual level in EDHS data that is why we didn't do the CAR model to visualize the random effects with covariates.

- Why bother reporting the un-adjusted odd ratios? THis seems pointless to me, as you end up adjusting them anyway. Like wise, you do not need to report the weighted n in your table 1, just the %'s

**Authors’ response**: Thank you, reviewer. We accepted the comments and we removed the COR and the weighted n in table 1. (See the revised manuscript)

- Finally, you need to specify a hypothesis, there are no real research questions or testable hypotheses specified.

**Authors’ response:** Thank you, reviewer. The research questions in this study were

1. Whether the spatial distribution of abortion is random or not? Answered by Global spatial autocorrelation test (Moran’s Index)
2. Where are the significant hotspot areas of abortion in Ethiopia? Answered by SaTScan analysis
3. What are the factors that are significantly associated with abortion? Answered by GLMM (mixed-effect logistic regression analysis)

**Reviewer #3**

This is an interesting and well written paper documenting clustering of abortion in Ethiopia. The importance of the topic is well framed in the introduction, and the investigators use appropriate methods to draw conclusions.

**Authors’ response:** Thank you, reviewer.
My only suggestion is that the manuscript receive another read through to make minor edits to sentence structure for clarity.

**Authors’ response:** Thank you, reviewer. We extensively edit sentence structures and grammar with the help of language experts. (See the revised manuscript)

**Reviewer #4**

The authors present an interesting examination of the spatial distribution of abortion in Ethiopia using demographic and health survey data. The methods are appropriately used, with one question about whether the residuals were spatially autocorrelated in the model.

**Authors’ response:** Thank you, reviewer. We analyzed global spatial autocorrelation using Moran's Index and was significant. It revealed that the spatial distribution of abortion was non-random with Global Moran's I 0.06 (p*<*0.001) (significant spatial dependence). (See the revised manuscript)
1. Lines 25-27, line 63, line 65, line 67, line 69 and throughout. The authors should specify that “unsafe abortion” rather than “abortion” is a major cause of maternal mortality and a public health concern. Right now the two concepts are conflated.

**Authors’ response:** Thank you, reviewer. We have corrected as unsafe abortion in the document but in EDHS the data was collected as abortion it was not separately recorded as unsafe and safe abortion. (See the revised manuscript)
2. Line 33, line 36. Can the authors define abortion in this population? Is this the percentage of women interviewed who ever had an abortion? Or the percentage of previous pregnancies that ended in abortion?

**Authors’ response:** Thank you, reviewer. For this study, we define "abortion as the percentage of previous pregnancy that ended in abortion". (See the revised manuscript)
3. Throughout the authors talk about the rate of abortion and the prevalence of abortion. The authors need to define each, which I believe will have different meanings and interpretations. Are these prevalence of women ever having an abortion, prevalence of pregnancies ending in abortion, or what?

**Authors’ response:** Thank you, reviewer. You are right the rate of abortion and prevalence of abortion is different in meaning and interpretations. When we say the rate of abortion it is defined as the number of pregnancy ended in abortion per 1000 pregnancy whereas the prevalence of abortion is defined as the percentage of abortion per 100 pregnancy. For this study, we had reported the prevalence of abortion. (See the revised manuscript)

4. The authors should limit significant digits on figure 3. 0-5%, 5-15%, 15-28%, 28-50%.

**Authors’ response:** Thank you, reviewer. We have modified the maps as you recommend us. (See the revised manuscript)
5. The authors did not present the results from the global moran’s I of the residuals of their regression model. Was this non-significant, indicating that the model explained the spatial variance in the outcome? Or did the authors need to adjust their approach to account for spatially correlated data?

**Authors’ response:** Thank you, reviewer. We didn't do the spatially weighted regression since there is no covariate collected at the EA level. We did only the Global spatial autocorrelation, spatial interpolation, and SaTScan analysis.
6. For figure 5, I presume this is the LISA? Please indicate

**Authors’ response:** Thank you, reviewer. Figure 5 was Local indicators of spatial autocorrelation (LISA) using Getis Ord Gi statistics of hotspot analysis to identify significant hotspot areas and significant cold spot areas of abortion. As per the editors' comment, we had removed Figure 5 since we used SaTScan analysis to identify significant hotspot areas of abortion and this is very informative from a public health perspective.

7. The authors should limit significant digits on Figure 6 in a same manner as comment 4.

**Authors’ Response:** Thank you, reviewer. We have corrected by limiting significant digits. (See the revised Figure)
8. For figure seven I suggest using a single color for their hot spots identified (presuming these are all hotspots). They’re all significant.

**Authors’ response:** Thank you, reviewer. We have modified the figure as you suggest and we have clipped within the study area. (See the revised Figure)
9. I didn’t see a good subsection in the methods on the variables considered for the regression model. This needs to be better explained. In the table there was no urban/rural, which I would expect to be a significant factor.

**Authors’ response:** Thank you, reviewer. We have stated the variables considered for the regression model in the revised manuscript. The place of residence was one of the significant factors associated with abortion and it is already found in the regression table. (See the revised manuscript)
10. I think the authors would benefit from a copyeditor for the English.

**Authors’ response:** Thank you, reviewer. We had extensively edit the grammar and sentence structure with the help of Language experts. (See the revised manuscript)
