# Supplementary material for: Spatial distribution and determinants of abortion among reproductive age women in Ethiopia, evidence from Ethiopian Demographic and Health Survey 2016 data: Spatial and mixed-effect analysis
Source: PLoS One. 2020 Jun 29;15(6):e0235382. doi: 10.1371/journal.pone.0235382 (PMC7323954; doi:10.1371/journal.pone.0235382)
Supplement: Attachment — Submitted filename: Point by point response.docx [file pone.0235382.s003.docx]

**PLOS ONE**

**Point by point response for editors/reviewers comments**

**Manuscript title:** Spatial distribution and determinants of abortion among reproductive-age women in Ethiopia, Evidence from Ethiopian Demographic and Health Survey (EDHS) 2016 data: Spatial and Mixed-effect analysis

**Manuscript ID:**  PONE-D-19-30532R1

**Dear editor/reviewer.**

Dear all,

We would like to thank you for this constructive, building, and improvable comments on this manuscript that would improve the substance and content of the manuscript. Further, the details of changes we made is shown by track changes in the supplementary document attached. The manuscript language was checked by language professionals and we follow journal guideline. **Response to Editors comments**

1. Specifically, please address the issues raised in the attached pdf file. Additionally, the manuscript has numerous grammatical errors that render it not suitable for publication in its current form. Therefore, I strongly recommend that you get it reviewed and thoroughly edited by a native English speaker.

**Authors’ response:**  Thank you Editor for the comments. We had extensively revised the manuscript. The grammar and editorial errors was extensively edited, and reviewed thoroughly with the help of language experts working at university of Gondar (See the revised manuscript).
